# An unusually delayed presentation of massive haematemesis following stab injury to the chest

**DOI:** 10.1093/jscr/rjab081

**Published:** 2021-04-06

**Authors:** Abidemi A Adesuyi, Oladele O Situ

**Affiliations:** General Surgery Unit, Department of Surgery, National Hospital Abuja, Garki, Abuja, Nigeria; General Surgery Unit, Department of Surgery, National Hospital Abuja, Garki, Abuja, Nigeria

## Abstract

Penetrating thoracoabdominal injury is a common presentation in the trauma resuscitation room with the possibility of a myriad of injuries which may involve thoracic and abdominal viscera. Management is usually operative however non-operative management is a possibility especially following knife stab injuries when compared with gunshot injuries. Clinical presentation will depend on the injured organ, extent of injury and time from injury to presentation. Unusual presentation of delayed haematemesis is a possibility with injury to the stomach, however, due to its rarity, a high index of suspicion with emergency surgery will help to mitigate the fatal consequences that may follow. This type of presentation is not documented in the available on-line literature which portends the importance of this paper. This case highlights the possibility of this clinical presentation and importance of early management to improve patient outcome.

## INTRODUCTION

Massive haematemesis can be an unusual, delayed presentation following penetrating thoracoabdominal injury with a knife stab more so when this occurs 4 days post-injury. A thorough search through on-line literature has shown that no case has been reported to occur after 48 h of injury with primary cause being laceration of the stomach. Based on the foregoing, we report the first case of delayed presentation of massive haematemesis following thoracoabdominal knife stab injury in the absence of major vascular injury.

The index patient had an urgent exploratory laparotomy with intraoperative findings of ~500 ml of clotted blood in the peritoneum, 2.5-cm perforation on the anterior wall of the stomach and 4-cm laceration on the posterior surface of the liver. This case exemplifies the challenges of misdiagnosis and non-operative management of penetrating thoracoabdominal injury.

## CASE REPORT

A 43-year-old obese man presented at our trauma centre complaining of bleeding from a laceration on the right hemithorax just below the nipple following a knife stab 30 min prior to presentation. Primary survey following the ATLS® protocol showed a respiratory rate (RR) was 22 cyc/min, oxygen saturation level (SPO_2_) was 96% on room air, pulse rate (PR) was 103 bpm, blood pressure (BP) was 131/79 mmHg. Bleeding from his wound was controlled. He had a 12-month history of uncontrolled diabetes mellitus.

**Figure 1:**
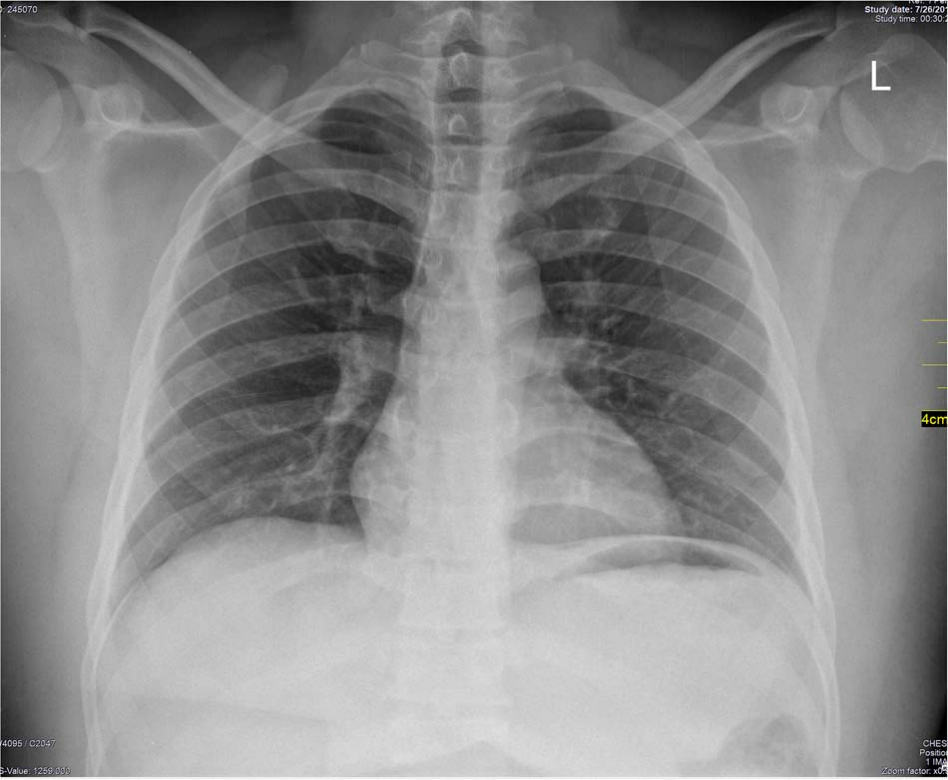
Chest X-ray PA view; normal chest X-ray.

**Figure 2:**
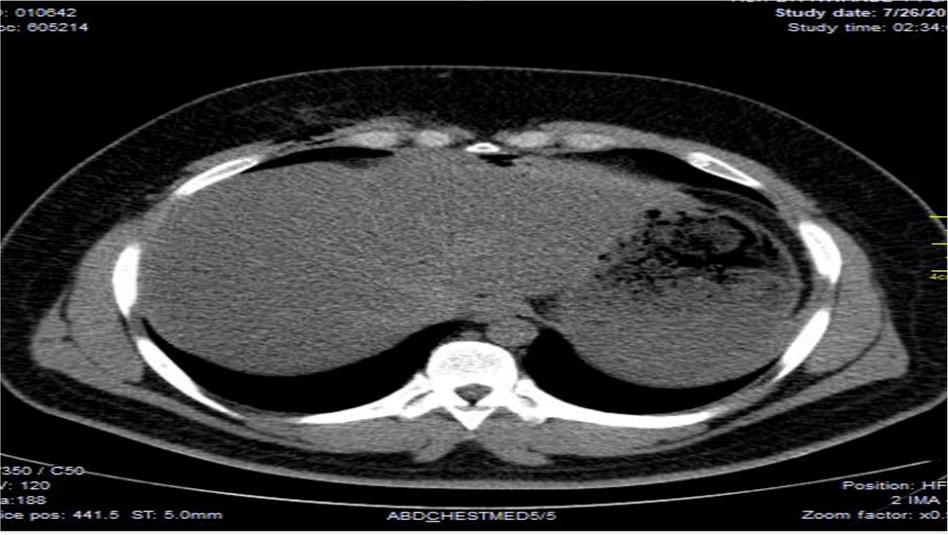
Image showing air lucencies in the right intercostal muscles and subcutaneous tissue around the lower right parasternal region.

**Figure 3:**
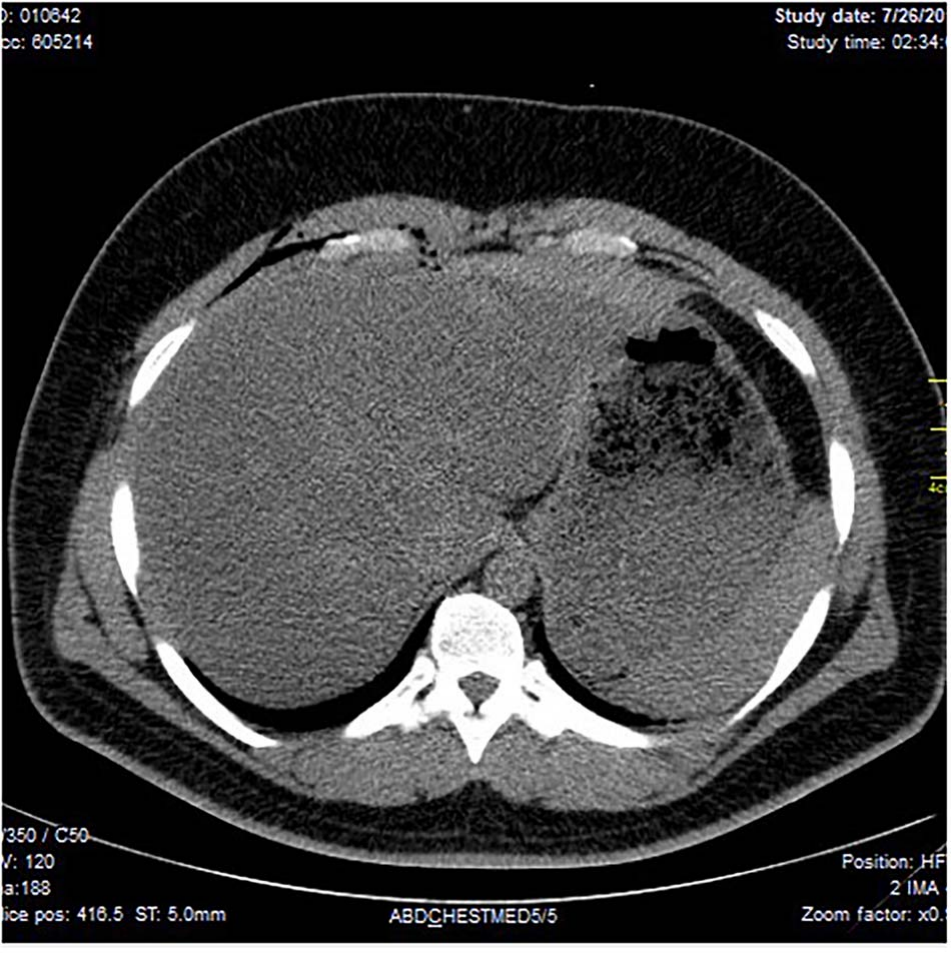
Showing lucent air densities tracking through the right intercostal muscles and abutting on the liver in the epigastrum with associated subcapsular haematoma in the left lobe of the liver.

**Figure 4:**
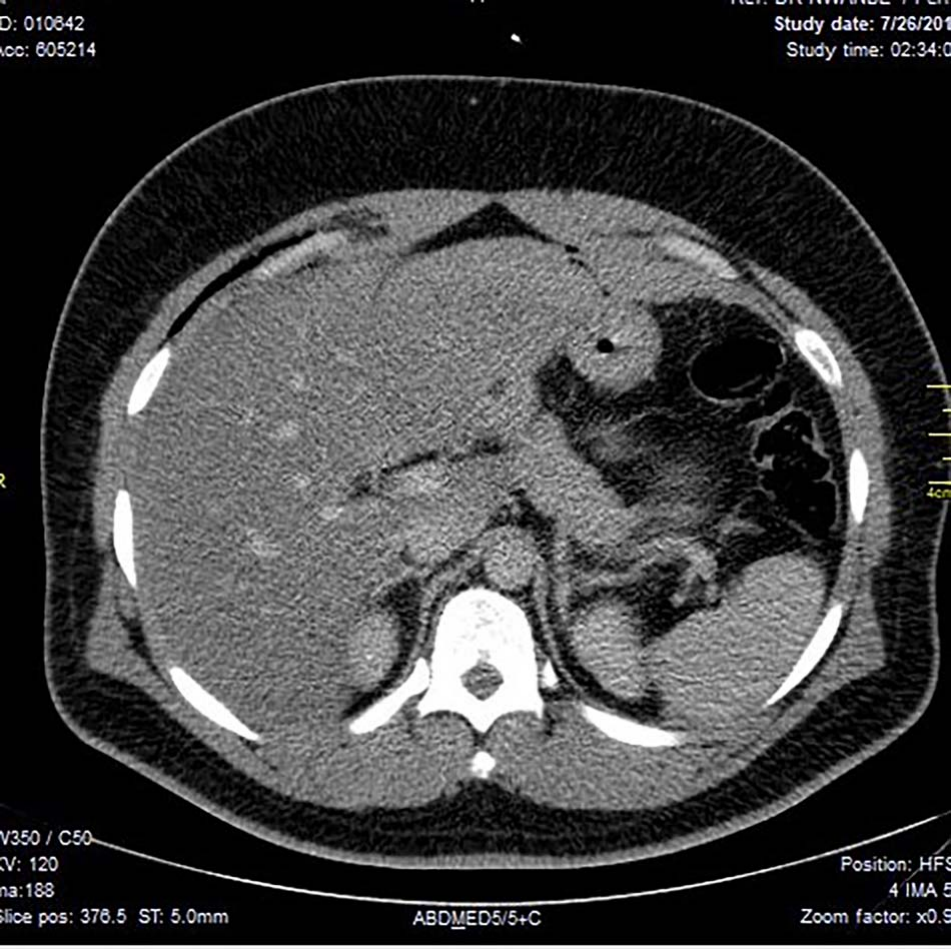
Image shows lucent air densities tracking through the subcapsular haematoma in the left lobe of the liver anterior to the pylorus of the stomach.

Chest X-ray was normal as shown in Fig. 1. Focused abdominal sonography for trauma (FAST) scan showed mild left perihepatic collection necessitating a thoracoabdominal computerized tomography (CT)-scan which showed external oblique muscle laceration with intermuscular air lucencies and grade 2 hepatic injury as shown in Figs 2–4. Other blood work-up were normal. No other injury was found on secondary survey. His wound was explored under local anaesthesia, primarily repaired, and he was discharged after 24 h of close observation.

He however re-presented 4 days later with a 3-h history of haematemesis (three episodes with each estimated to be ~500 ml). He was lethargic, diaphoretic, dehydrated and pale. His airway was clear. RR was 28 cyc/min and SPO_2_ was 98% on O_2_ by facemask, PR was 140 bpm and BP was 141/63 mmHg. Chest examination was unremarkable but vague tenderness was elicited in the left hypochondrial region with no demonstrable ascites. Further systemic examination was unremarkable.

His bedside FAST showed presence of fluid in Morrison’s pouch and pelvis. Blood investigations are as shown in Table 1.

**Table 1:** Blood investigation results

	Values	Reference
Hb	8.2 g/dl	13–18 g/dl
PCV	25%	40–54%
WBC	14 × 10^9^/l	4.8–10.8 × 10^5^/l
Platelets	271 × 10^9^/l	140–400 × 10^5^/l
Random blood sugar	17 mmol/l	<11.1 mmol/l
Cl^−^	100 meq/l	96–108 mmol/l
Na^+^	138 meq/l	135–150
K^+^	3.9 meq/l	3.5–5.3
Urea	8.6 mmol/l	2.5–6.4
Creatinine	85 μmol/l	44–100 mcmol/l
AST	32 IU/l	≤38 IU/l
ALT	25 IU/l	≤40 IU/l
ALP	82 IU/l	≤104 IU/l
GGT	38 IU/l	≤38
Total Bilirubin	8.9 μmol/l	<17.1 mcmol/l
Conjugated Bilirubin	4.7 μmol/l	<4.3 mcmol/l
Total protein	62 g/dl	60–80 g/l
Albumin	26 g/dl	30–50 g/l

A diagnosis of massive upper gastrointestinal (GI) bleeding from penetrating thoracoabdominal injury was made. He was resuscitated with crystalloids, blood products, analgesics and glucose control with glucose potassium insulin infusion (GKI) regimen. He had exploratory laparotomy with findings of about 500 ml of clotted blood in the peritoneal cavity, 4 and 2.5-cm lacerations on the posterior surface of the liver and anterior wall of the stomach, respectively. He had primary repair of the injuries.

He was admitted postoperatively into the ICU before being stepped down to the ward. Postoperatively, he was managed for deep surgical site infection, turbulent hyperglycaemia and new onset hypertension. He was discharged on 17th postoperative day.

## DISCUSSION

Penetrating injury to the chest following knife stab is a common presentation to the trauma centre in civilian setting with most patients being haemodynamically stable at presentation unlike when compared with gunshot injuries [1]. Stab injury below the nipple suggests possibility of thoracoabdominal injury.

Though cases of significantly delayed haematemesis following traumatic diaphragmatic hernia have been described [2, 3], the presentation of massive haematemesis beyond 48 h following thoracoabdominal stab injury with gastric laceration is rare.

Definitive diagnosis of an associated intra-abdominal injury is preferably confirmed by abdominal CT scan [4] in the haemodynamically stable patient while the unstable patients will usually undergo an exploratory laparotomy following a FAST/extended FAST exam. Occasionally, diagnostic trauma laparoscopy may be required to rule out occult diaphragmatic and viscera injuries [5]. Abdominal ultrasound has been reported to have a sensitivity of 65% and specificity of 98% with an accuracy of 88% in patients with penetrating abdominal injury [6]. Accuracy of CT in detecting solid intra-abdominal organ injury and retroperitoneal haematoma is higher when compared with gastric injury which has a sensitivity and specificity of 80 and 58%, respectively [7].

The paradigm of management of low-velocity torso penetrating injury has gradually shifted to initial conservative care in haemodynamically stable patients without altered consciousness or gross evisceration [8]. Some authors advocate video laparoscopy to rule out occult injuries [9]. The patient had an initial thoracoabdominal CT that showed a grade 2 hepatic injury, normal chest X-ray, remained stable necessitating non-operative management. He however, deteriorated on the fourth-day post-injury mandating an urgent exploratory laparotomy. Gastric injury usually results in large pneumoperitoneum or haemoperitoneum [10] however, this was obscured from the CT scan in this patient suggesting that conservative management of such patients may not be adequate. A serial helical-CT scan with clinical monitoring beyond 24 h might have detected the associated gastric injury before the fourth-day.

## CONCLUSION

Delayed life threatening haematemesis and sepsis may be the sequelae of gastric perforation following penetrating thoracoabdominal injury despite initial stable clinical state and this may be missed even with the use of contrast abdominal CT scan. Thus, a high index of suspicion must be kept in such patients with thorough radio-clinical monitoring and low threshold for surgery.

## CONFLICT OF INTEREST STATEMENT

None declared.
